# Machining Simulation of Novikov Profile Gear Models for Analysis and Computational Calculations

**DOI:** 10.3390/ma18051155

**Published:** 2025-03-05

**Authors:** Paweł Fudali, Tadeusz Markowski, Jacek Pacana

**Affiliations:** Faculty of Mechanical Engineering and Aeronautics, Rzeszow University of Technology, al. Powstancow Warszawy 12, 35-959 Rzeszow, Polandtmarkow@prz.edu.pl (T.M.)

**Keywords:** gears, gearboxes, FEM, CAD systems, Novikov profile, tooth profile

## Abstract

The paper presents the process of preparing gear models with an original profile for numerical analyses. We used solid modeling reflecting the gear cutting using the enveloping generation method. A script was prepared in AutoCAD to enable automatic simulation of the material removal process and obtain precise gear models. The analyzed gears had geometry based on Novikov’s engagement; however, during the tool design, the tooth profile was adjusted due to the incorrect values of certain parameters present in the standards. Gears models with a circular-arc tooth profile created in this process were used for finite element method (FEM) calculations in ANSYS. An analysis of the contact pattern for the loaded gearbox was conducted. The stress state for the analyzed gear transmission with the adjusted tooth profile was also determined.

## 1. Introduction

In the case of gears, wheels with an involute profile are the most common solution, which has influenced the automation of their design and manufacturing process. In many popular CAD systems, modules have been implemented to automatically generate models of gears with involute tooth profiles. This profile has been an area of research and analysis for many years, but there now appears to be little scope for improvement. The most important disadvantages of involute gearing are the high pressures on the tooth flank surfaces and the high tooth slip speeds [1,2]. Therefore, gear design solutions with new or known but less explored tooth contours are constantly sought.

Available computer-aided design (CAD) programs usually allow the generation of gears only with an involute tooth profile. Therefore, when using a different tooth profile, it is necessary to prepare accurate models of gears individually using standard functions available in CAD programs [3,4,5]. However, it is often impossible to obtain sufficiently accurate tooth profiles in this way, especially on the tooth root profile. The solution to this problem is to make a gear model by reproducing the process of cutting the gear ring using the enveloping generation methods.

Many authors have already presented models resulting from machining simulations for cylindrical gears [6,7,8] and bevel gears [9,10,11], but these publications concerned the involute profile of teeth. The authors’ method presented here concerns gears with a circular-arc profile. It can also be used for any tooth profiles, including cycloidal and convexo-concave [12,13,14].

Here, the method in question is presented using the Novikov profile as an example, since this profile, as opposed to the involute, provides better mating conditions in the mesh area [15,16]. The advantages of gears with a circular-arc tooth line had already been recognized by British and American designers at the beginning of the twentieth century, but the initial designs did not meet the durability requirements and were sensitive to changes in axle spacing [6,17,18]. Novikov introduced significant changes to the geometry of wheels of this type, presenting his profile with two engagement lines in the 1969 GOST-15023 standard. Subsequent modifications to this profile were aimed at increasing strength or improving mating conditions [19,20,21]. A special tooth profile recommended for use on materials with a hardness greater than 35HRC was also designed and is described in detail in GOST 30224-1996 [22]. Contemporary research [23,24,25] shows that the potential resulting from the use of a circular-arc profile in gears is not yet fully exploited, and new design solutions are still being sought.

The simulation of the machining of CAD gear models proposed in this article concerns gears with a Novikov profile. The analyzed models accurately reproduce the tooth profile that is obtained during the hobbing of real gears. The high quality of the models obtained in this process entitles them to be used in numerical FEM calculations and kinematic analyses. They have geometric features that allow for the use of finite element models of any type, shape, or size in the discretization process.

The presented method applies to gears with a Novikov profile, but it is universal and can be used for different tooth outlines. The limitations are only due to the general rules of gear meshing geometry. The simulation can use a tool (CAD model) with a different profile and simulate machining for an individually adopted tooth line angle. In this way, it is possible to quickly prepare a lot of models differing in the selected parameters and obtain detailed strength and durability analyses. Attempts have been made to automate the process of modeling gears with Novikov profiles, as there are no commercial programs for the modeling and analysis of gears with atypical, new, or modified contours. The search for non-standard design solutions in the field of tooth cooperation conditions in order to increase their durability is related by the authors [26,27]. Currently, such research is advisable due to the development of digitally controlled manufacturing methods and the availability of new materials to extend the range of use of gear transmissions.

Therefore, an analysis concerning the modification of the geometry of this profile has been carried out and described, a methodology for making accurate solid models has been developed, and a preliminary strength analysis using FEM has been carried out.

## 2. Geometry

The basic characteristic of a concave–convex mesh is the cooperation of the convex tooth surface of one wheel with the concave surface of the other wheel. The radii of curvature of the meshing teeth differ slightly, with the radius of the concave surface being greater than that of the convex surface. In such a profile, there is engagement through a tooth contact ratio and therefore the gears must be made as helical with a tooth helix angle of βmax = 10° ÷ 24° [28]. However, compared to gears with involute teeth, Novikov gears with the same geometrical features and parameters and made of the same materials carrying 1.5 to 1.7 times the load [2,18]. In addition, for the circular-arc profile, there is no geometric slippage, and the cooperation consists of the rolling of the teeth over each other in the axial direction, in a manner similar to the reciprocal rolling of two cylinders.

**Figure 1:**
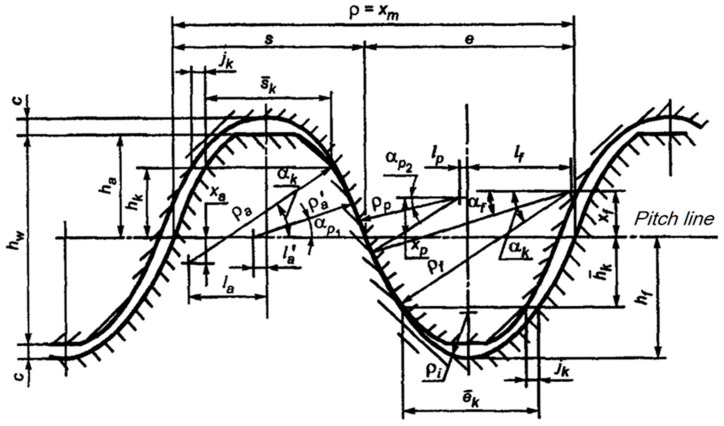
Scheme of a Novikov basic rack profile with two lines of action (TLA) according to the standard GOST 30224-96 [22], *ρ*_q_ = 0.05 mm, l_p_ = 0.05083 mm, s_k_ = 0.98086, x_a_ = 0.2, x_p_ = 0.32363.

In this study, the Novikov profile, which has two lines of contact and is presented in the 1996 GOST 30224 standard, has been taken into account. The advantage of this profile is that both mating wheels with a common modulus m_n_ can be made with one set of tools. The reference profile for the Novikov profile is shown in Figure 1, and the parameter values for modules smaller than 3.15 mm are given in Table 1.

Based on the profiles presented, the planar tool profiles and solid models of the gear cutting tools used in the simulation process were made in AutoCAD.

**Figure 2:**
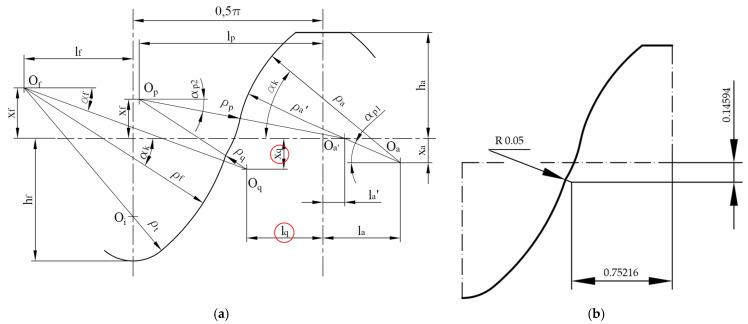
Correction of geometric parameter values: (**a**) profile according to GOST 20224-96 [18] with erroneous dimensions marked; (**b**) corrected profile with correct values of changed parameters.

When attempting to draw a tool profile, for the Novikov profile, according to the dimensions given in the relevant standard, it proved impossible to obtain a continuous profile. The problem was that there was no tangency between some of the profile curves, so it was impossible to connect them smoothly. This problem is most likely due to the rounding off of the values given in the standard for the parameters x_q_ = 0.14586 mm and l_q_ = 0.75213 mm. However, the error in the parameters defining the position of the center of the radius was too large for the program to automatically connect the subsequent sections of the profile with an arc of radius *ρ*_q_ = 0.05 mm. Due to the need to maintain a continuous tooth side profile, it was assumed in further consideration that there was tangency between the profile curves. The value of the radius *ρ*_q_ was retained, while the values of the dimensions x_q_ and l_q_ were changed from those given in the standard. Figure 2a shows the reference profile of the standard according to GOST 30224-96 with the incorrect parameters highlighted. Figure 2b shows the corrected profile with the corrected values of the coordinates of the center of the radius *ρ*_q_, which were used during the tests.

Once the correct gear geometry had been determined, the models were prepared in AutoCAD corresponding to the geometry of the gear tools. In a first step, the corrected tooth profile was drawn according to the recommendations of the standard and corrected according to the author’s adjustments. The modulus m_n_ = 2.5 mm and the number of teeth of the pinion gear z_1_ = 21 and of the gear wheel z_2_ = 35 were adopted. The profile was then supplemented with additional edges to form a closed sketch corresponding to the two teeth of the pinion gear.

Finally, a command was used to draw the profile in the defined direction, which was deviated from the axis perpendicular to the plane of the profile by an angle corresponding to the inclination of the tooth line of the designed wheel.

## 3. Gear Modeling

The 3D CAD modeling of gears can be realized by various analytical and geometric methods. One of the commonly used methods is the mathematical representation method, where tooth profiles are modeled, which are directly graphical forms of often complex functions describing the tooth profile [29,30]. However, gears designed in this way are difficult to manufacture on conventional machine tools. Therefore, in the present work, it is assumed that wheel models will be obtained by simulating machining. This will allow the obtained solutions to be repeated for real gears and made using conventional machining processes.

**Figure 3:**
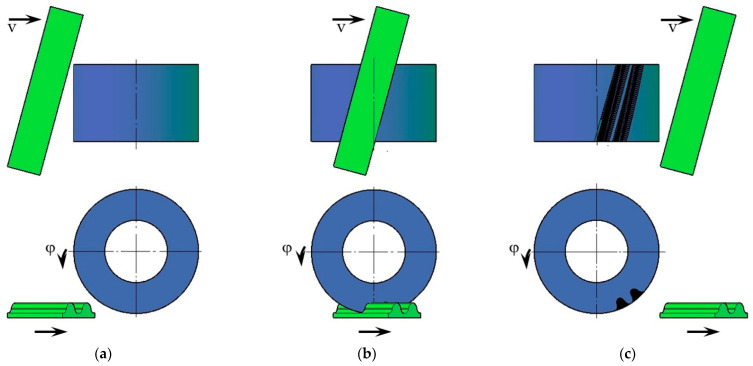
Stages of gear cutting simulation in AutoCAD: (**a**) before contact; (**b**) geometry subtraction; (**c**) after cutting.

In the present case, it was assumed that the Sunderland method [18,31] would be simulated to make solid gear models. The entire process was carried out in Autodesk AutoCAD, which was also used to make the tool and wheel model. The two models were set in a position derived from the gear geometry and parametrically linked to each other. In the initial position (Figure 3a), the tool and wheel models were not in mutual contact. The wheel by a defined angle *φ* was linearly realized, and the tool was moved by a value corresponding to the length of the arc bounded by this angle on the rolling diameter of the gear. In the next step, the tool was subtracted from the gear wheel using Boolean operations (Figure 3b). This step was repeated many times until the complete profiles of two consecutive intertooth notches were obtained (Figure 3c). Due to the significant number of repeated operations, a macro was developed to automate the tasks performed for the described calculation step.

The models of the gear and pinion were prepared with the same tool and using the same script to simulate the actual machining of the gear.

**Figure 4:**
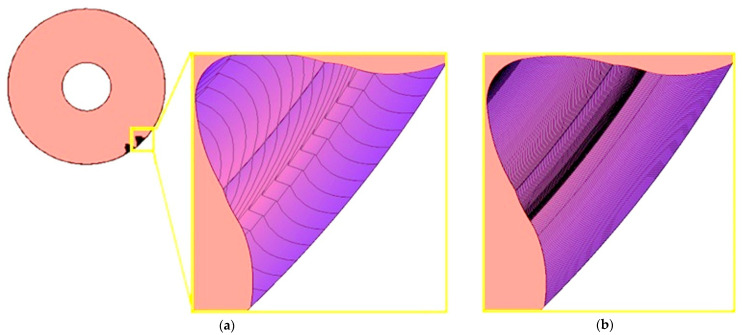
Example of simulation results for a uniform wheel rotation angle as follows: (**a**) *φ* = 1°; (**b**) *φ* = 0.1°.

As part of the study, a series of models was performed for different values of the angle of rotation of the machined wheel, which was taken successively at 1; 0.5; 0.2; 0.1 degrees per simulation step. Changing the value of the unit angle of the wheel rotation significantly affected the quality of the lateral surfaces obtained. A view of an enlarged section of the model obtained for the smallest and largest adopted angle value *φ* is shown in Figure 4. Visual evaluation of the sample results reveals very large discrepancies between the models. However, for angles of *φ* = 0.5° and *φ* = 0.2°, the differences in edge density are no longer so noticeable.

**Figure 5:**
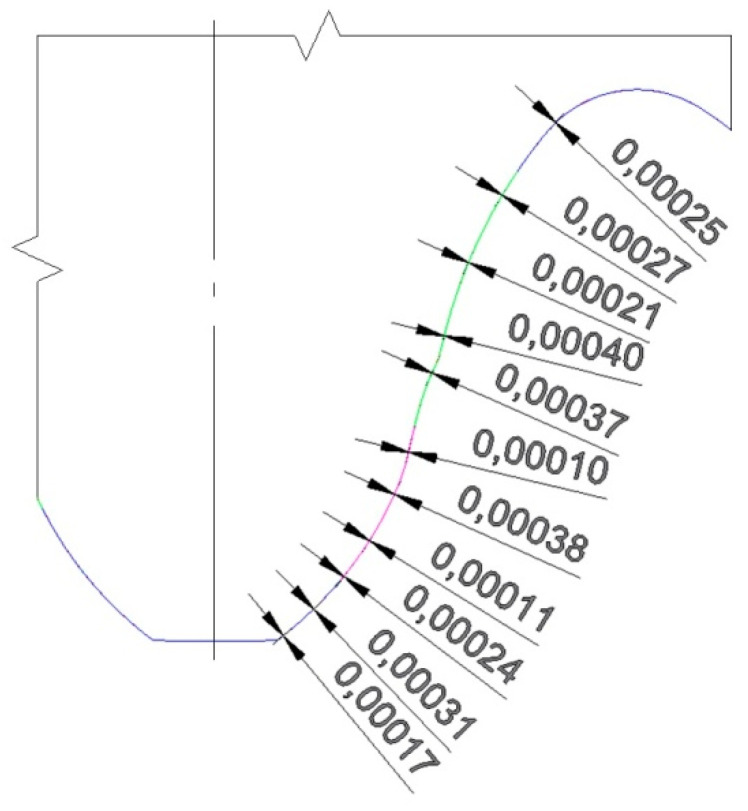
Distances between tooth profiles for the corrected profile, for a unit rotation angle value of *φ* = 0.1° and *φ* = 0.2°.

To determine the differences in the geometry of the models obtained, the tooth profiles obtained from the cutting simulations for different angles were compared with each other. A comparison of the obtained tooth profiles, in the face plane of the wheels, was also carried out in AutoCAD. The results for angles of *φ* = 0.1° and *φ* = 0.2° were very close to each other, as shown in Figure 5. The maximum difference in the relative distance between the profiles was 0.0004 mm, so it did not exceed the allowed deviations specified for a standardized tooth flank contour [32,33].

For larger values of unit wheel rotation angles, the inaccuracies were larger, so these models were not used for accurate tooth contact analysis and strength calculations. The gear machining carried out ran automatically, but varied significantly in duration depending on the assumed angle. For the most accurate models with an angle of *φ* = 0.1°, the simulation time was 12 h and was three times longer than for an angle of *φ* = 0.2°. It should be noted that the simulation of notching only two consecutive notches was assumed, so the processing time for a full circle would be much longer. To avoid this, it would be advisable to duplicate the tooth profile obtained by using the circular array command in CAD.

With the insignificant differences in the tooth profile, it is appropriate to continue using models created with a unit rotation angle of *φ* = 0.2°.

## 4. Smoothed Solid Models

Although the machining simulation carried out makes it possible to obtain very accurate solid models of gears, the resulting tooth side surfaces are not a single smooth surface. Such a model causes many difficulties and is inconvenient to use in numerical strength and kinematic analyses. Often, such calculations are performed using the finite element method (FEM), and the overly complex geometry of batch models can cause mesh generation problems or calculation errors.

Therefore, the gear models obtained from the simulation should be simplified while not compromising their high quality. To this end, all surfaces previously created on the side of the tooth should be replaced by a single smooth and continuous surface. The limitations of various CAD programs usually do not allow such an operation to be carried out automatically. Therefore, it is necessary to use special functions that allow the definition of complex surfaces spanning guide curves. The SIEMENS NX program was chosen for this task because, in addition to a very powerful module for creating and editing surfaces, it has tools for evaluating their quality.

**Figure 6:**
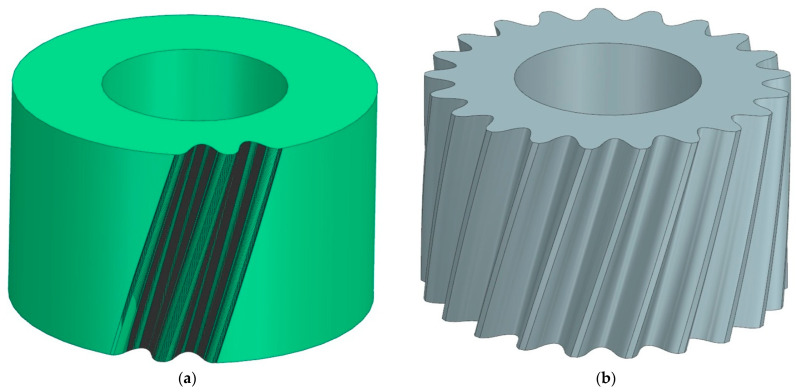
Modeling stages of a smoothed pinion model with the number of teeth z_1_ = 21: (**a**) model obtained after machining simulation; (**b**) smoothed model with smooth tooth side surfaces.

**Figure 7:**
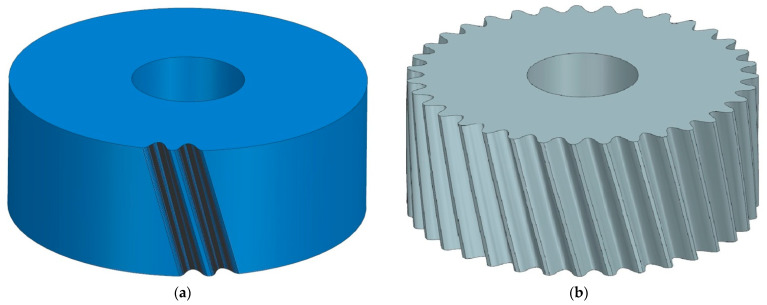
Modeling stages of a smoothed model of a gear wheel with the number of teeth z_2_ = 35: (**a**) model obtained after machining simulation; (**b**) smoothed model with smooth tooth side surfaces.

To create a smoothed tooth side surface, a base profile and guide curves had to be prepared. The profile was read from the gear model obtained from the machining simulation (Figure 6a and Figure 7a) and was replaced in NX by a compound curve that describes all the vertices located in the front section of the tooth profile. The profile was obtained by defining complex curves on the edges of the model. This procedure allowed us to create smooth surfaces that describe the sides of the tooth. These surfaces were then copied around the perimeter of the wheels in an amount corresponding to the number of teeth, and after applying some basic functions of the NX, the final gear models were obtained. The finished gear models with the corrected profile are presented in Figure 6b and Figure 7b.

As part of the research work, full gear models were also prepared, in which the flanking surfaces of all teeth were a collection of many surfaces obtained through machining simulations. Due to the large size of these files and the huge number of surfaces, working with them in CAD was very slowed down and the application of arbitrary commands was very difficult. Also, exporting these accurate models to other programs and further use in, e.g., FEA analysis caused many errors, or sometimes it was even impossible to use them due to excessive demands on the computer’s operating memory.

## 5. Comparison of Smoothed Models and Those Generated by Machining Simulation

**Figure 8:**
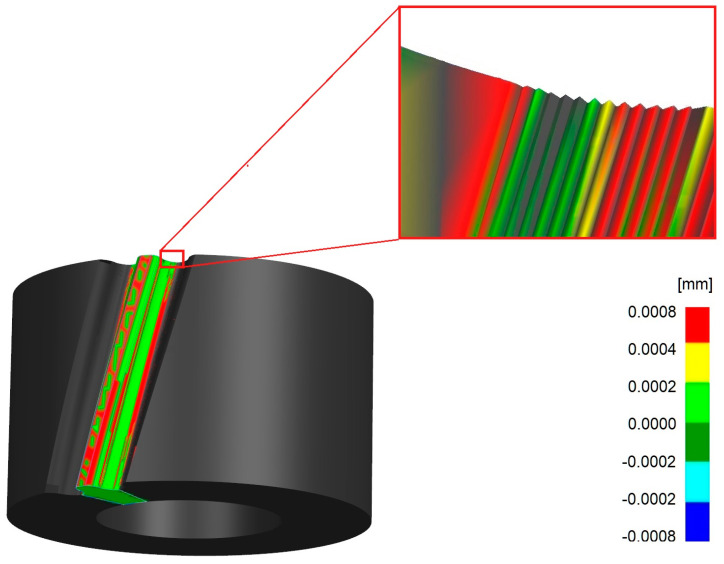
Map of the surface distance of the exact model generated by the machining simulation and the smoothed model.

Using the NX tools available in the Analysis module, an accuracy inspection of the previously prepared smoothed models was carried out. A comparison was made between the exact model resulting from the machining simulation and the surface formed on the side of the tooth in the smoothed model. The limiting acceptable value of the distance between the surface of the exact model and the smoothed surface was assumed to be 0.001 mm. This is the smallest value of the dimensional tolerances assumed in the machining and inspection of actual gears. Most of the inspected models met these requirements, and only the models for which the unit rotation angle *φ* = 1° was assumed in the machining simulation had a part of the surface outside the specified tolerance. In NX, the results of the model comparison are presented as a color map on the surface of the selected part as in Figure 8. This allows for a general evaluation, but also for a more detailed analysis of selected parts. Figure 8 also presents an enlarged section of the model near the base of the tooth. The colors corresponding to the correct distances of the compared surfaces resemble a staircase distribution, which are precisely the result of shaping the exact model in the machining simulation process.

In the comparative analysis in NX, it is also possible to obtain information on what percentage of the surface of the tested model is inside the assumed tolerance field. Since virtually all the prepared models fell within the preconceived tolerance range of 0.001 mm, the tolerance field was narrowed to ±0.0002 mm for the purpose of the analysis, and the results summarized for all gears are given in Table 2.

The NX program analysis module was also used to inspect the tooth profile in the frontal plane that passes through the center of the gear width. Similarly, the tooth profiles were evaluated in the normal plane defined in the center of the tooth line length.

**Figure 9:**
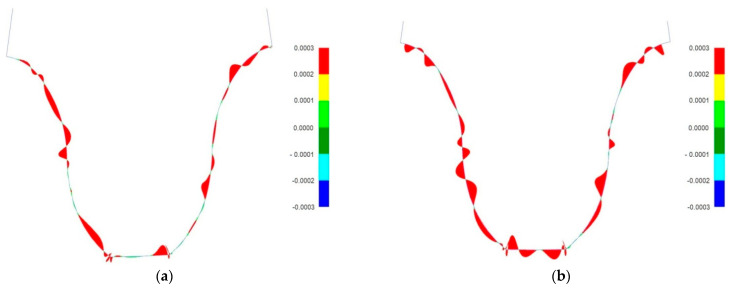
Results of comparison of tooth profiles of the tested wheels with corrected profiles in the following: (**a**) normal plane section; (**b**) transverse section.

Figure 9 shows an example graph obtained in NX when comparing tooth profiles for models obtained by simulated machining with a smoothed model. Also, for this analysis, a very restrictive tolerance field of ±0.0002 mm was adopted to clearly show the method. Results that exceed this value are colored red.

Clearly, a better plane fit occurs in the normal plane than in the frontal plane. For none of the tested wheel models, however, were the deviations between the tested surfaces greater than 0.001 mm. For each of the analyzed cases, it is possible to check, in a text file saved automatically on the computer, detailed information about the fit of the profiles. Among other things, this gives the number of points checked, the range of the tolerance field, the maximum distance error, and the average distance error or the angle error. Among all models tested, the highest distance value f = 0.000968294 was read for a pinion model with a unit machining angle of *φ* = 1°. This confirms the high geometric convergence of the tooth lateral surfaces of both types of models.

The comparative analyses performed confirmed that it is possible to obtain accurate solid models of gears in CAD programs. The proposed method of smoothing the tooth profile did not result in a loss of accuracy in terms of the tooth profile. The presented course of action is particularly advisable when it is necessary to prepare models for numerical strength analyses. The obtained models are full and complete, and at the same time, they are continuous so they allow any definition of the finite element mesh.

## 6. FEM Calculations

Smoothed models of gears with corrected profiles, prepared according to the given recommendations, were used for further analysis using FEM. Numerical calculations for the stress distribution and contact pattern were carried out in ANSYS 2024R1. Only fragments of the models were used, which reduced computer memory requirements and computational time. This method of using FEM calculations with simplified gear models is successfully used by many researchers [34,35]. The material properties, loads and degrees of freedom of the models, contact conditions, and expected results were also determined for the analyzed gears. The computational models were subjected to a discretization process using hexagonal finite elements of type SOLID186. A total of more than 320,000 finite elements were created for a computational model consisting of a pinion and gear. The calculations were realized in one design step with a constant torsional moment load of 200 Nm. In the simulation, the pinion was forced to rotate completely by 40° in single steps of 1°, which allowed the cooperation of several pairs of teeth to be traced. Revolute joints were used, one for the inner cylindrical surface of the pinion and another for the gear. To transfer the rotation of the gears, the deformable frictional contact was created. The tooth side faces of the pinion were the source (contact) and the tooth flanks of the gear were the target. In the contact section, the element types CONTA174 and TARGE170 were used with a coefficient value of 0.1.

**Figure 10:**
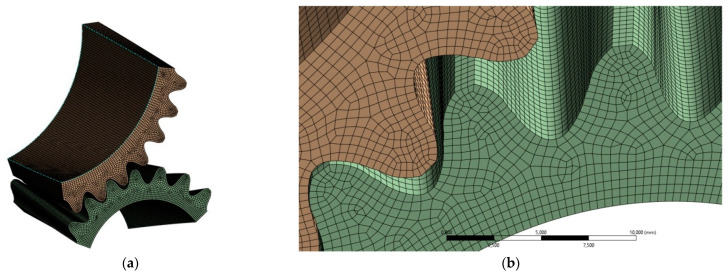
Finite element mesh view of smoothed gear models in ANSYS: (**a**) overall view of models; (**b**) enlargement of the ring gear area.

Because the surfaces defining the model are smooth and continuous, it was possible to choose any type of finite element and its number and size in each area of the computational models in a standard way. By using smoothed models, an even and correct mesh was obtained throughout the model, guaranteeing accurate solutions (Figure 10).

The possibility of the arbitrary arrangement of mesh nodes on the side of the tooth also gives the possibility of reading values at specific points on the tooth surface. This makes it possible to verify the numerical results with those obtained by analytical or experimental methods by comparing them at specific points on the profile [36].

**Figure 11:**
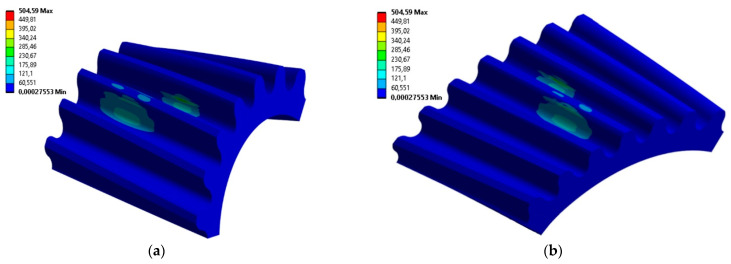
The von Mises stress distribution for an angle of *φ* = 0.1° on the following: (**a**) pinion; (**b**) gear wheel model.

All previously prepared smoothed gear models were processed for FEM calculations in the same way. Thus, the same boundary conditions for material and load, as well as type size and component arrangement, were assumed. The smoothed models for different unit angles *φ* of wheel rotation during machining simulation differed only slightly, so the computational models were also very similar. The results obtained for the distribution of stresses and the contact pattern did not differ by more than a few percent, which can be taken as an assumed inaccuracy of the calculations. Thus, presented in Figure 11 are examples of the obtained results for the analyzed profile. The presentation in the form of a map of the distribution of von Mises stresses applies to models with an angle of *φ* = 0.1°. The results correspond to the moment when the highest stress values occurred on the surface of both the gear wheel and the pinion models.

**Figure 12:**
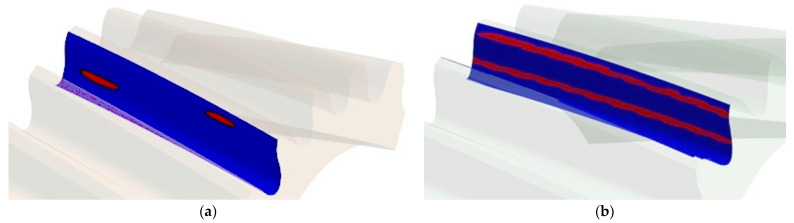
Areas of wheel mating on the pinion model with a corrected profile: (**a**) instantaneous contact pattern; (**b**) contact pattern.

Based on the same calculations, the contact pattern under load tooth contact analysis (LTCA) can be determined in ANSYS. Example results for a pinion with a corrected profile are presented in Figure 12a. The stage of mating was chosen when three teeth are simultaneously in the mesh. There are two contact areas on the middle tooth, since the analyzed tooth profile was modeled based on a Novikov profile with two lines of contact. The surface pressure values read for the two gears remaining in the mesh are at a similar and safe level slightly exceeding 670 MPa.

Based on the calculations, it is also possible to determine the areas where contact between gears occurs during the entire mesh cycle. Figure 12b presents the contact pattern for one tooth of a pinion with the analyzed profile.

**Figure 13:**
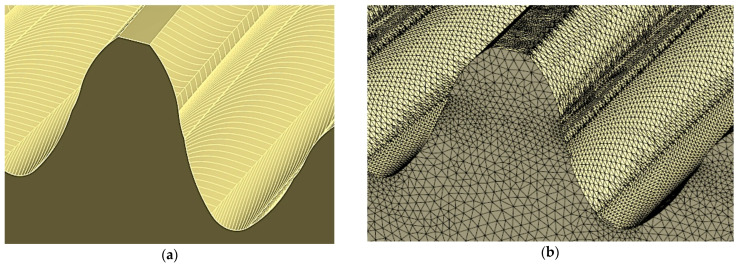
Partial view of the exact model of the gear wheel obtained from the machining simulation: (**a**) before the finite element mesh was generated; (**b**) with the finite element mesh.

Numerical calculations were also performed for designed gears with the analyses obtained directly after the machining simulation process. Due to the considerably higher workload and the much longer time concerning the preparation of models and performing the calculations, only models with a single wheel rotation angle of 0.2° at machining were analyzed. These exact models had tooth sides composed of many surfaces, so the generation of the finite element mesh was much more complicated in this case. It was not possible to generate element sizes and shapes arbitrarily because the nodes of the mesh had to be located at the vertices of the surface describing the tooth side. Even if a more accurate edge division was enforced in the gear wheel model, the position of the vertex nodes could not be changed. Therefore, in the case of accurate models, it was impossible to arbitrarily change the size of the mesh and adjust it properly for the contact surfaces of two mating wheels. It was impossible to define a mesh with a larger distance between nodes than the distance of the vertices defining the surface model. Therefore, the models obtained in the highly accurate machining simulation had more than 4500 thousand tetrahedral finite elements of the SOLID187 type. This is several times more than those for a smoothed model. Furthermore, it was impossible to freely choose the type of finite elements, since the ANSYS program only allowed for the use of tetrahedral elements (Figure 13). Apart from the differences in defining the finite element mesh, all the input conditions were adopted exactly the same for the numerical calculations.

**Figure 14:**
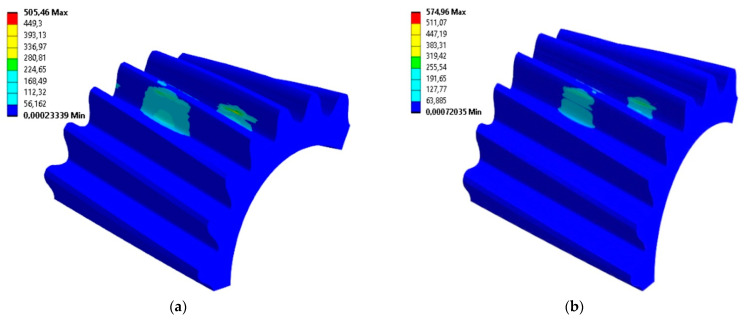
The von Mises stress on the pinion gear for the following: (**a**) the model obtained from simulated machining with a unit rotation angle b = 0.2°; (**b**) the smoothed tooth profile.

**Figure 15:**
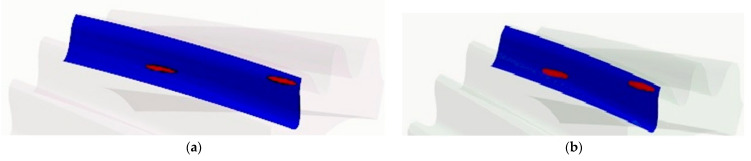
Instantaneous contact pattern on the pinion gear for the following: (**a**) the model obtained from simulated machining with unit rotation angle b = 0.2°; (**b**) the smoothed tooth profile.

Comparing the results for stress values at the same points on the tooth surface and comparing the maximum values with each other showed that the differences between the results obtained for the exact and the smoothed models are not significant. The maximum values of the von Mises stresses read on the side of the tooth for the smoothed model of the gear wheel were 467 MPa, and for the exact model were 458 MPa. Additionally, the maximum values of surface pressures identified in the area of tooth engagement differed by only 2% and were about 678 MPa (both for the smoothed and exact profiles). Figure 14 compares with each other the results of the FEM calculations of the von Mises stresses for smoothed and fine models in the same stage of cooperation. The position of instantaneous contact patterns for the same models and at the same moment of cooperation was also analyzed (Figure 15).

A comparison of the results obtained for the exact and the smoothed profiles showed no significant differences in their quality or detail. The results for the smoothed models show high repeatability, continuity, and a more regular distribution on the models. This is due to the uniform distribution and proportional shape of the finite elements used to describe the computational models. The preparation time of the smoothed models for numerical calculations, the calculations themselves, and the processing of the results are at an acceptable level. It is therefore advisable to use smoothed models in the numerical analyses of gears with circular-arc contours.

## 7. Summary and Conclusions

The presented machining simulation method makes it possible to generate gear models using commercial AutoCAD 2024 software. As a result of the modeling process carried out, which reproduces actual enveloping generation methods, very accurate solid models are obtained. However, too much detail causes problems with their visualization and modification, and limits the possibility of using them in calculations and numerical simulations. Therefore, this publication also proposes a method for converting accurate models into smoothed models using functions available in popular CAD programs. The resulting gears are accurate enough to be used directly in computer analysis and simulations. An additional advantage of using smoothed models is the ease of their modification and discretization, as well as the wide range of available methods for presenting and verifying the results of the numerical calculations carried out. Therefore, it is recommended to use the described modeling method, especially for gears with a non-evolved tooth profile.

The methodology for the preparation of smoothed models is discussed using the example of gears with a circular-arc tooth profile. Using the prepared script for the automatic simulation of machining, a number of models differing in the unit angle of rotation of the tool *φ* were made. The comparison of the results of the FEM calculations in terms of the von Mises stresses and the contact pattern for the exact and smoothed models did not show significant differences. This confirms the usefulness of the described method and the high accuracy of the prepared solid gear models.

The corrected Novikov profile considered in the analysis, despite its advantages, is relatively rarely used in gear construction. This is mainly due to the low availability of tools, lack of knowledge of gear manufacturing technology, and the fact that it is still not studied in detail. The profile still leaves many opportunities for improvement in terms of durability and functionality, which becomes possible through the use of modern numerical analysis. However, all numerical calculations should be verified by other testing methods to confirm their increased load capacity and strength. For those selected, the most advantageous design solutions of the analyzed gears with circular-arc profiles, bench tests were also planned.

The presented methodology provides opportunities to more easily prepare models and to perform numerical calculations for further computational design work on gears with a Novikov profile.

## Figures and Tables

**Table 1:** The main parameters of Novikov’s profile for the module m < 3.15 mm.

Parameter	GOST 30224-96 [mm]
*ρ*_a_	1.38
*ρ*_f_	1.76
*ρ*_i_	0.85
l_a_	0.64
l_f_	0.90128
h_a_	0.875
h_f_	1.01163
h_k_	0.59154
S	1.46797
e	1.67362
c	0.15
α_k_	35°
j_k_	0.1

**Table 2:** Overview of the number of surfaces located in the tolerance field of 0.0002 mm.

Accuracy of the Reference Model	Surfaces in the Tolerance Field [%] for the Corrected Profile	
	Pinion Gear	Gear Wheel
*φ* = 1°	63.134	69.764
*φ* = 0.5°	78.995	83.457
*φ* = 0.2°	94.724	89.175
*φ* = 0.1°	96,428	86.924

## Data Availability

The original contributions presented in this study are included in the article. Further inquiries can be directed to the corresponding author.
